# RETRACTED: Gupta et al. Chickpea Peptide: A Nutraceutical Molecule Corroborating Neurodegenerative and ACE-I Inhibition. *Nutrients* 2022, *14*, 4824

**DOI:** 10.3390/nu17172754

**Published:** 2025-08-26

**Authors:** Neha Gupta, Sameer Quazi, Saurabh Kumar Jha, Mohammad Khursheed Siddiqi, Kanika Verma, Swapnil Sharma, Rizwan Hassan Khan, Sameer Suresh Bhagyawant

**Affiliations:** 1 School of Studies in Biotechnology, Jiwaji University, Gwalior 474011, Madhya Pradesh, India; 2 GenLab Biosolutions Private Limited, Bangalore 560043, Karnataka, India; 3 Department of Biomedical Sciences, School of Life Sciences, Anglia Ruskin University, Cambridge CB1 1PT, UK; 4 School of Health Sciences, University of Manchester, Manchester M13 9P, UK; 5 SCAMT Institute, ITMO University, 197101 Saint Petersburg, Russia; 6 Department of Biotechnology, School of Engineering & Technology (SET), Sharda University, Knowledge Park-III, Institutional Area, Greater Noida 201310, Uttar Pradesh, India; 7 Department of Biotechnology Engineering and Food Technology, Chandigarh University, Mohali 140413, Punjab, India; 8 Department of Biotechnology, School of Applied & Life Sciences (SALS), Uttaranchal University, Dehradun 248007, Uttarakhand, India; 9 Molecular Biophysics and Biophysical Chemistry Group, Interdisciplinary Biotechnology Unit, Aligarh Muslim University, Aligarh 202002, Uttar Pradesh, India; 10 Department of Pharmacy, Banasthali Vidyapeeth, Banasthali 304022, Rajasthan, India

The *Nutrients* journal retracts the article “Chickpea Peptide: A Nutraceutical Molecule Corroborating Neurodegenerative and ACE-I Inhibition” [1] cited above.

Following publication, concerns were brought to the attention of the Editorial Office regarding the authorship list and origins of this study [1].

Adhering to our standard procedure, an investigation was conducted by the Editorial Office and Editorial Board, which was unable to confirm the accuracy of the authorship list nor the origins of the study. Further concerns relating to the integrity of Figure 12 and the accuracy of the underlying data remained unresolved due to a lack of verifiable raw material. Consequently, the Editorial Board has lost confidence in the reliability of the findings and decided to retract this publication [1], as per MDPI’s retraction policy (https://www.mdpi.com/ethics#_bookmark30, accessed on 6 July 2025).

This retraction was approved by the Editor-in-Chief of the *Nutrients* journal.

The authors did not agree to this retraction.
